# Utility of spherical human liver microtissues for prediction of clinical drug-induced liver injury

**DOI:** 10.1007/s00204-017-2002-1

**Published:** 2017-06-13

**Authors:** William R. Proctor, Alison J. Foster, Jennifer Vogt, Claire Summers, Brian Middleton, Mark A. Pilling, Daniel Shienson, Monika Kijanska, Simon Ströbel, Jens M. Kelm, Paul Morgan, Simon Messner, Dominic Williams

**Affiliations:** 1 Investigative Toxicology, Department of Safety Assessment, Genentech, Inc., 1 DNA Way, South San Francisco, CA 94080 USA; 2 Drug Safety and Metabolism, AstraZeneca, Alderley Park, Macclesfield, Cheshire, SK10 4TG UK; 3 Discovery Sciences, AstraZeneca, Alderley Park, Macclesfield, Cheshire, SK10 4TG UK; 4 Cambridge Science Park, Cambridge, Cambridgeshire CB4 0WG UK; 5 Non-clinical Biostatistics, Product Development, Genentech, Inc., 1 DNA Way, South San Francisco, CA 94080 USA; 6 InSphero AG, Wagistrasse 27, CH-8952 Schlieren, Switzerland

**Keywords:** DILI, Spheroid culture, Microtissue, Hepatocyte, Hepatotoxicity, Drug discovery

## Abstract

Drug-induced liver injury (DILI) continues to be a major source of clinical attrition, precautionary warnings, and post-market withdrawal of drugs. Accordingly, there is a need for more predictive tools to assess hepatotoxicity risk in drug discovery. Three-dimensional (3D) spheroid hepatic cultures have emerged as promising tools to assess mechanisms of hepatotoxicity, as they demonstrate enhanced liver phenotype, metabolic activity, and stability in culture not attainable with conventional two-dimensional hepatic models. Increased sensitivity of these models to drug-induced cytotoxicity has been demonstrated with relatively small panels of hepatotoxicants. However, a comprehensive evaluation of these models is lacking. Here, the predictive value of 3D human liver microtissues (hLiMT) to identify known hepatotoxicants using a panel of 110 drugs with and without clinical DILI has been assessed in comparison to plated two-dimensional primary human hepatocytes (PHH). Compounds were treated long-term (14 days) in hLiMT and acutely (2 days) in PHH to assess drug-induced cytotoxicity over an 8-point concentration range to generate IC_50_ values. Regardless of comparing IC_50_ values or exposure-corrected margin of safety values, hLiMT demonstrated increased sensitivity in identifying known hepatotoxicants than PHH, while specificity was consistent across both assays. In addition, hLiMT out performed PHH in correctly classifying hepatotoxicants from different pharmacological classes of molecules. The hLiMT demonstrated sufficient capability to warrant exploratory liver injury biomarker investigation (miR-122, HMGB1, *α*-GST) in the cell-culture media. Taken together, this study represents the most comprehensive evaluation of 3D spheroid hepatic cultures up to now and supports their utility for hepatotoxicity risk assessment in drug discovery.

**Electronic supplementary material:**

The online version of this article (doi:10.1007/s00204-017-2002-1) contains supplementary material, which is available to authorized users.

## Introduction

Drug-induced liver injury (DILI) continues to be a leading cause of attrition during drug development, withdrawal post-marketing, and cautionary/restrictive labeling (Watkins 2011). Hepatotoxicity risk is difficult to predict based on the various etiologies that encompass DILI (Chalasani et al. 2008), with unknown factors driving patient susceptibility towards hepatic stress and injury (Chalasani and Bjornsson 2010), coupled with the poor concordance of preclinical species to identify human hepatotoxicants in vivo (Olson et al. 2000). However, retrospective analysis over the past 50 years has identified several epidemiologic risk factors associated with DILI that include but are not limited to physicochemical properties of the drug, dose and disposition, and signals in a battery of in vitro assays (Dambach 2014). For example, high daily dose (>100 mg) and lipophilicity (log *P* > 3) (Chen et al. 2013), significant hepatic metabolism (>50% dose) (Lammert et al. 2010), and being a substrate for CYP450 enzymes (Yu et al. 2014) have all been positively associated with clinical incidence of DILI. In concordance with high daily dose, compounds whose total plasma exposure, in particular *C*_max_, were greater than 1.1 μM, were associated with DILI from those that were not (Shah et al. 2015). A drug’s potency to inhibit the transporters of bile-acids (bile salt export pump (BSEP, ABCB11) and multidrug-resistance protein-4 (MRP4, ABCC4)) has been shown to correlate with human hepatotoxicity (Kock et al. 2014; Morgan et al. 2010), which was increased when corrected for the total steady state plasma concentration (Morgan et al. 2013). Similarly, the ability of a drug to adversely affect mitochondrial function (O’Brien et al. 2006; Porceddu et al. 2012) was associated with increased risk for DILI, which was further enhanced when considering other risk factors such as BSEP inhibition and dose/exposure (Aleo et al. 2014; Shah et al. 2015).

Lastly, drug-induced cytotoxicity in hepatic cell lines (Gustafsson et al. 2014; O’Brien et al. 2006; Shah et al. 2015; Xu et al. 2008) or primary-plated hepatocytes (Schadt et al. 2015) has also been associated with human hepatotoxicity, especially when considering dose or exposure (Schadt et al. 2015; Shah et al. 2015). Depending on the endpoints and compound sets employed, these assays generally experience sensitivities between 50 and 70% and specificities of 70 and 90% to identify human hepatotoxicants (Dambach 2014; Schadt et al. 2015). These high-to-medium throughput testing platforms have been proposed for incorporation in early phase drug development, in combination with preclinical in vivo studies to aid in optimizing compounds with favorable safety attributes. These include the use of cell-based imaging assays in HepG2 cells or human hepatocytes (Garside et al. 2014; O’Brien et al. 2006; Persson et al. 2013; Tolosa et al. 2012; Xu et al. 2008), or cell viability assessment in SV-40 transformed human liver epithelial (THLE) cells (Dambach et al. 2005; Gustafsson et al. 2014). As hepatotoxicity has been proposed to occur as a result from multiple mechanisms for many drugs, some workers have used multi-parametric analysis in a single cell type (Garside et al. 2014; O’Brien et al. 2006; Persson et al. 2013; Tolosa et al. 2012; Xu et al. 2008) and others a panel of individual cell based and bile-acid transporter inhibition assays (Aleo et al. 2014; Schadt et al. 2015; Shah et al. 2015; Thompson et al. 2012) to predict hepatotoxicity retrospectively. However, few of the models contain the full complement and functionality of metabolic enzymes and transporters present in human hepatocytes in vivo (Gustafsson et al. 2014; Wilkening and Bader 2003), which is also true with plated primary human hepatocytes (PHH) that rapidly loose liver phenotype and CYP450 activity in traditional monolayer cultures (Rodriguez-Antona et al. 2002). These factors significantly limit the ability of these platforms to detect metabolite-induced cytotoxicity as well as the effects of the parent drug and its metabolites on bile-acid homeostasis/intrahepatic cholestasis and mitochondrial impairment.

Recent advances in more physiologically relevant hepatic in vitro models have created promising tools to enhance prediction of hepatotoxicity in drug discovery. These emerging platforms include, but are not restricted to, plated micro-patterned co-cultures of hepatocytes with stromal fibroblasts (Khetani and Bhatia 2008; Khetani et al. 2013), three-dimensional (3D) bioprinted liver tissues comprised of several hepatic cell types (Ma et al. 2016; Nguyen et al. 2016), and 3D spheroid cultures either as mono-culture or co-culture with hepatic non-parenchymal cells (NPC) (Bell et al. 2016; Messner et al. 2013). In most cases, these systems displayed enhanced metabolic activity, hepatocellular phenotype, and stability in culture, previously not attainable with traditional hepatic cell line or hepatocyte models [refer to review (Godoy et al. 2013)]. For example, micro-patterned co-cultures of primary rat or human hepatocytes and stromal fibroblasts demonstrated that long-term (e.g., 7 days) treatment in this system outperformed conventional cultures to identify hepatotoxicants when assessing GSH depletion, albumin and urea secretion, and cell viability assessment for a 45-compound test set (Khetani et al. 2013). Previous studies using this platform demonstrated increased CYP450 activity and improved stability of liver phenotype over time in culture compared to monocultures of primary hepatocytes (Khetani and Bhatia 2008), which was hypothesized to be driving in part the increased sensitivity towards hepatotoxicants (Khetani et al. 2013). Similarly, hepatic spheroid models have garnered interest as additional tools to aid in predicting DILI (Bell et al. 2016; Hendriks et al. 2016; Messner et al. 2013). The 3D spheroid models have been reported to maintain metabolic activity and viability for up to 28 days in addition to the presence of canicular membrane structures (Bell et al. 2016; Hendriks et al. 2016; Messner et al. 2013). Recent published work suggests that long-term treatment (28 days) in liver spheroid cultures increased sensitivity for detection of a panel of five drugs known to cause DILI clinically (Bell et al. 2016). However, a thorough retrospective assessment of known DILI-positive and DILI-negative compounds in a 3D liver spheroid model is lacking. To this end, we investigated cytotoxicity of 110 marketed drugs comprised of both DILI positives (63%) and negatives (37%) in 3D human liver microtissues (hLiMT) that are made up of primary human hepatocytes and non-parenchymal cells (e.g., Kupffer cells) for repeat-dose long-term treatment. For comparison, we also assessed cytotoxicity for the identical compound set in plated PHH from the same human donor used to prepare the hLiMT. The work presented here provides the most comprehensive evaluation of 3D liver spheroids up to now for retrospective prediction of clinical hepatotoxicity. Using drug-induced cytotoxicity as an endpoint, hLiMT assay experienced increased sensitivity and specificity to identify known human hepatotoxicants in relation to plated PHH. Together with recently published studies, this work supports 3D hepatic spheroid models as promising tools to aid in hepatotoxicity risk assessment during drug discovery.

## Materials and methods

### Reagents and chemicals

Cryopreserved PHH, primary human non-parenchymal cells (NPCs), InVitroGro HT™ thawing media, InVitroGro CP™ plating media, and 1% Torpedo™ Antibiotic Mix were obtained from BioreclamationIVT, Baltimore, MD, USA. CellTiter-Glo^®^ Assay was obtained from Promega, Madison, WI, USA. 3D InSight™ Human Liver Microtissues, GravityTRAP™ plates, 3D InSight™ Human Liver Maintenance Medium (hLiMM) AF and hLiMM TOX were obtained from InSphero AG, Schlieren, Switzerland. Microclime^®^ lids were obtained from Labcyte, Sunnyvale, CA, USA. BioCoat collagen I 384-well plates were obtained from Corning Life Sciences, Corning, NY, USA. All pharmaceutical drugs were provided by AstraZeneca Compound Management, AstraZeneca R&D, Macclesfield, United Kingdom, or Sigma Aldrich, St. Louis, MO, USA). All other reagents were obtained from Sigma-Aldrich Company Ltd (Poole, Dorset, UK or St. Louis, MO, USA).

### Cytotoxicity assessment in 2D primary human hepatocytes

Cryopreserved PHH (lot IZT) were thawed in InVitroGro HT™ thawing media at 37 °C, pelleted, and resuspended. Viable hepatocytes were counted by Trypan blue exclusion and plated in black-walled, BioCoat™ collagen 384-well plates at 13,000 cells/well in InVitroGro CP™ plating media supplemented with 1% Torpedo™ Antibiotic Mix and 5% fetal bovine serum and incubated overnight for 18 h. Cells were then treated with compounds for 48 h diluted in InVitroGro HI™ incubation media containing 1% Torpedo Antibiotic Mix, 10% fetal bovine serum, and 1% DMSO. Cell viability was determined at the end of the experiment by CellTiter-Glo^®^ Assay following the manufacturers protocols. Luminescence was determined on an EnVision™ Muliplate Reader (PerkinElmer, Waltham, MA, USA), and data were normalized to vehicle (1% DMSO) control wells. Inhibition curves and IC_50_ estimates were generated by non-linear regression of log-transformed inhibitor concentrations (8-point serial dilutions including vehicle) vs. normalized response with variable Hill slopes, with top and bottom constrained to a constant values of 100 and 0, respectively (GraphPad Prism™, GraphPad Software, La Jolla, CA, USA). The highest concentration tested for each compound was either the 100× the total clinical maximal plasma concentration (*C*_max_) for the individual compound or the limit of solubility in 1% DMSO in media if the 100× margin could not be achieved.

### Cytotoxicity assessment in 3D human liver microtissues

All spheroid hLiMT used in this study were 3D InSight™ Human Liver Microtissues (InSphero AG, Schlieren, Switzerland) and produced according to a patent-pending protocol (WO2015/158777A1) using the hanging-drop method. GravityTRAP™ plates with single hLiMT in each well were covered with Microclime^®^ lids and incubated at 37 °C in a humidified 5% CO_2_ cell-culture incubator in BSA-free 3D InSight™ hLiMM TOX medium. PHH (lot IZT) in co-culture with NPCs (lot RHV) were used to assess the cytotoxicity of all compounds listed, except for Dexamethasone. Additionally, other hepatocytes lots: IZT, OFA, SSR, and EBP, co-cultured with different NPC lots: RHV, JJB, ZAR, and QGU were used to assess donor-dependent cytotoxicity for selected compounds.

Compound treatment started 6 days after seeding and lasted for 14 days. Re-dosing of the hLiMT was performed after 5 and 9 days from initial dosing. Seven serial dilutions of 200X or 100X compound stocks in DMSO and the vehicle controls were aliquoted and frozen for each dosing. At the day of treatment, aliquots were diluted to working concentration with hLiMM TOX. Working concentration of acetaminophen, cycloserine, ethotoin, flucloxacillin and levocarnitine, along with corresponding dilutions were prepared directly for each dosing in hLiMM TOX. For a subset of compounds, 5–6 days of treatment was performed. For these studies, compounds were re-dosed on day 3 and the experiment was concluded at day 5 or 6. The concentrations tested for each compound were identical to those employed for the PHH cytotoxicity assessment outlined above.

Viability of hLiMT was determined at the end of the experiment with CellTiter-Glo^®^ 2.0 Cell Viability Assay and luminescence were read on a SPARK™ 10 M plate reader (Tecan, Männedorf, Switzerland). Data from compound-treated microtissues were normalized to the respective vehicle controls (0.5 or 1% DMSO) cultured on the same GravityTRAP™ ULA plate. The IC_50_ values were calculated in GraphPad Prism™ using identical methods listed above for PHH IC_50_ value estimations.

### Compound list and DILI categorization of pharmaceuticals

The 110 drugs evaluated for cytotoxicity in vitro were each assigned to one of five categories as described previously (Garside et al. 2014), using information extracted from the peer reviewed scientific literature and from data contained in product labels. The details of the drugs and their categories are listed in Table 1 and Supplementary Table S1. Twenty-three have been withdrawn from clinical use due to DILI, or have been given Black Box warnings for DILI in the US product labels, and were categorized as severity category 1 ‘‘Severe clinical DILI.’’ Twenty-three drugs have been associated with acute liver failure in humans, but have not been withdrawn or given DILI Black Box warnings, and were categorized as severity category 2 ‘‘High clinical DILI concern.’’ Twenty-three drugs have been reported to cause symptomatic liver injury, but not liver failure, and were categorized as severity category 3 ‘‘Low clinical DILI concern.’’ Sixteen drugs have been associated with raised serum levels of alanine aminotransferase and other enzymes indicative of drug-induced liver dysfunction, but have not been reported to cause symptomatic DILI, and were categorized as severity category 4 ‘‘Enzyme elevations in clinic.’’ The remaining 25 drugs have not been associated with evidence of liver dysfunction and were categorized as severity category 5 ‘‘No DILI.’’ Eighty-one of the 110 drugs have been assigned to either High, Low or No DILI concern classes by other investigators, who considered the clinical severity of DILI reported in the clinic and labeling approved by US FDA (Chen et al. 2011, 2016). This information is summarized in Supplementary Table S1 as ‘‘LTKB DILI classification.’’ For binary classification of the compound set into compounds positive for clinical DILI and those without, any compound in DILI classes 1–3 were considered DILI+ve and categories 4–5 were determined to be DILI–ve.

**Table 1:** Overview of compounds test set with their corresponding DILI severity category

DILI severity category	Compounds
1: Severe clinical DILI (*N* = 23)	Amiodarone, Benaxoprofen, Benzbromarone, Bosentan, Danazol, Dantrolene, Felbamate, Flutamide, Ketoconazole, Lapatinib, Methotrexate, Nefazodone, Perhexiline, Sitax(s)entan, Stavudine, Sudoxicam, Sunitinib, Tienilic Acid, Tolcapone, Troglitazone, Trovafloxacin, Valproic Acid, Ximelagatran
2: High clinical DILI concern (*N* = 23)	Amodiaquine, Atorvastatin, Azathioprine, Carbamazepine, Celocoxib, Clozapine, Diclofenac, Flucloxacillin, Imipramine, Indomethacin, Itraconazole, Levofloxacin, Meloxicam, Naproxen, Nimesulide, Nitrofurantoin, Paroxetine, Rosiglitazone, Simvastatin, Tacrine, Tamoxifen, Ticlopidine, Zileuton
3: Low clinical DILI concern (*N* = 23)	Acetaminophen, Acetylsalicylic Acid, Amitriptyline, Beta-Estradiol, Chlorpheniramine, Chlorpromazine, Clomipramine, Cyclophosphamide, Desipramine, Fluoxetine, Furazolidone, Metformin, Mitomycin C, Nifedipine, Penicillin V, Phenformin, Pimozide, Pioglitazone, Quinacrine, Rosuvastatin, Spectinomycin, Tretinoin, Verapamil
4: Enzyme elevations in clinic (*N* = 16)	Bumentanide, Buspirone, Cycloserine, Dabigatran, Dexamethasone, Entacapone, Ethotion, Felodipine, Fludarabine, Meclofenamate, Minoxidil, Nadolol, Nicardipine, Pargyline, Penbutolol, Theophylline
5: No DILI (*N* = 25)	Albuterol, Alendronate, Ambrisentan, Benserazide, Benztropine, Digoxin, Flavoxate, Flumazenil, Guanethidine, Hyoscyamine (Daturine), Indoramine, Levocarnitine, Liothyronine, Mecamylamine, Metergoline, Neostigmine, Orphenadrine, Oxybutynin, Phenoxybenzamine, Phentolomine, Procyclidine, Propantheline, Pyridostigmine, Streptomycin, Zomepirac

DILI severity category labeling according to Garside et al. (2014)

### Statistical methods for data: receiver operating characteristic analysis and likelihood ratio calculations

The objective of the statistical analysis was to compare the utilities of the PHH and hLiMT assays in terms of their ability to predict DILI-positive and -negative compounds. The 110 compounds common to both assays were analyzed (Table 1). Compounds were classified as either DILI positive (DILI+ve) or DILI negative (DILI−ve) (i.e., binary classification) using each assay IC_50_ [μM] and the ratio of the IC_50_ [μM] to total plasma *C*_max_ [μM] (referred also throughout the manuscript as an assay “margin of safety” (MOS) for each compound) as the classifier. Several practical thresholds spanning the range of IC_50_ or MOS values were used for classification. The sensitivity and specificity of each assay were calculated by comparing DILI positive/negative status as determined by assay IC_50_ or MOS threshold with known DILI status for each compound. In addition, the concordance of each assay with known DILI status was assessed using Cohen’s Kappa (Cohen 1960). Kappa is interpreted as follows: kappa = 1 signifies full agreement between assay classification and known DILI class, and kappa ≤ 0 signifies no agreement other than what would be expected by random chance. The *P* value tests the null hypothesis that kappa = 0. Concordance analysis using Cohen’s kappa goes beyond simple calculation of the proportion of agreement by accounting for the expected proportion of chance agreement, which depends on the number of DILI+ve and DILI−ve compounds present in the sample set. A first pass analysis consisted of removal of censored compounds—i.e., compounds without IC_50_ values obtained—prior to calculating sensitivity and specificity. Statistical evaluation involved using receiver operating characteristic (ROC) analysis (Altman and Bland 1994b) to determine a classification boundary between the two classes. The criterion for defining the discrimination threshold minimized the distance in the ROC curve from the perfect assay (sensitivity 100% and specificity 100%). The sensitivity and specificity were generated from a tenfold cross-validation of the classification model to avoid bias in using the data to both define the threshold and determine its characteristics. Statistical analysis was performed using R Version 3.0.1 (R Core Team 2013). For comparing the utility of each assay to identify hepatotoxicants from those that are not associated with clinical hepatotoxicity, we calculated the positive likelihood ratio (PLR) and negative likelihood ratio (NLR) based on the sensitivity and specificity estimates outlined above. Likelihood ratios represent the ratio of the probability of the specific test result for compounds associated with DILI to the probability of compounds that do not cause DILI. Likelihood ratios summarize sensitivity and specificity to characterize the utility of an assay for increasing certainty about a diagnosis and are less dependent on disease prevalence, which is important for low incidence events such as DILI. In addition, these parameters can be calculated directly from sensitivity and specificity estimates for tests that have binary results (Altman and Bland 1994a; Deeks and Altman 2004). In practice, a PLR value of 1 indicates no influence on the risk of disease, values between 2 and 5 indicate a small/moderate increase in probability, and values of 10 or greater indicate a large and often certain increase in the likelihood of disease. Similar interpretation is considered for NLR values, but inversely to PLR with values ranging from 1 to approaching 0.

## Results

### Comparison of drug-induced cytotoxicity in 2D plated primary human hepatocytes and 3D human liver microtissues

**Fig. 1:**
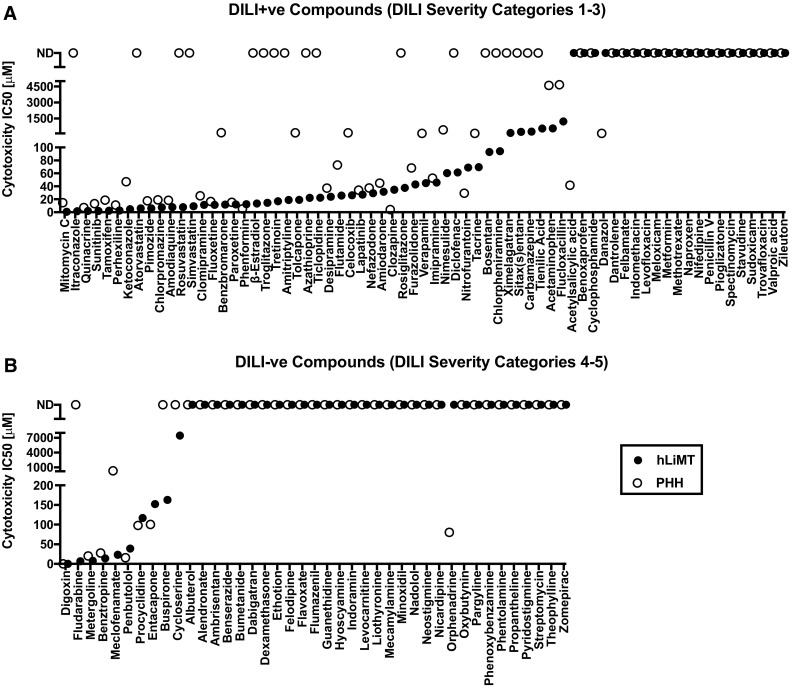
3D hLiMT were more sensitive to overall drug-induced cytotoxicity than 2D PHH. Cytotoxicity IC_50_ values for 110 drugs in PHH treated for 48 h (*open symbols*) and hLiMT treated for 14 days (*closed symbols*) for compounds classified as DILI+ve (DILI severity categories 1–3) (**a**) and DILI–ve (DILI severity categories 4–5) (**b**), respectively. Data represent IC_50_ value estimate for each compound listed on the *x*-axis. For compounds where no IC_50_ value converged, these were plotted at the *top* and classified as not determined (ND)

Drug-induced cytotoxicity, as measured by decreases in total cellular ATP content, of the 110 compounds listed in Table 1 was determined in both PHH treated for 48 h and in hLiMT treated for 14 d. The data from these studies are summarized in Supplemental Tables S-2 and S-3, respectively. Additionally, exemplary dose–response curves can be found in Supplementary Figure S1. The challenge faced when comparing these two datasets were that there were more IC_50_ values determined for the hLiMT in relation to the PHH for both DILI+ve and DILI–ve compounds. As depicted in Fig. 1a, IC_50_ values were not determined (ND) (e.g., IC_50_ value was greater than the highest dose tested) for 54% (37/69) and 33% (23/69) of the DILI+ve compounds assessed in PHH (open symbols) and hLiMT (closed symbols), respectively. The number of compounds without IC_50_ values increased to 80% (33/41) and 76% (31/41) determined in PHH and hLiMT (Fig. 1b) in the DILI−ve compound class, which was as expected from their clinical safety profile. In total, hLiMT detected more IC_50_ values (56/110) for the compound set than PHH (40/110) under these conditions, supporting that the hLiMT assay was more sensitive overall to drug-induced cytotoxicity than the plated PHH assay.

### Statistical analysis employing ROC assessments

**Fig. 2:**
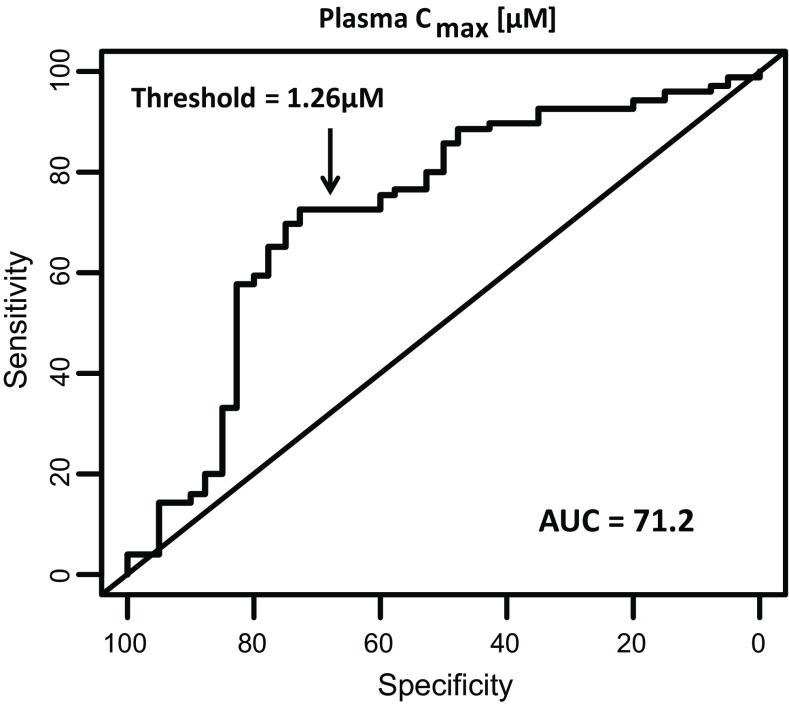
Optimized receiver operating curve (ROC) for total plasma concentration (*C*_max_) alone as predictor of clinical hepatotoxicity. Optimized ROC curve for total plasma concentration (*C*_max_) for 110 drugs associated with and without clinical hepatotoxicity. ROC curve was generated from total plasma *C*_max_ data for the test set and an optimized threshold (*in bold*) was determined

Dose and drug exposure, as measured by the total plasma *C*_max_ levels, has been shown to be associated with DILI (Chen et al. 2013; Shah et al. 2015). Accordingly, we asked what the predictive value of the *C*_max_ levels in the complete test set in Table 1 was in the absence of any assay cytotoxicity assessment. Assay sensitivity and specificity were optimized across the *C*_max_ values for the binary DILI classified compound set using ROC analysis. ROC curve analysis of the total plasma *C*_max_ (Fig. 2) demonstrated overall sensitivity and specificity of 72.5 and 73.2%, respectively, at an optimized threshold of 1.26 μM with the highest area under the ROC curve (AUC) value (72.1) of all the comparisons. The threshold was very similar to a recently published threshold of 1.1 μM total plasma *C*_max_ set as a predictor of hepatotoxicity for a test set of 125 commercial drugs (Shah et al. 2015). Using the fixed threshold of 1.26 μM, the PLR and NLR values for total plasma *C*_max_ as a predictor of hepatotoxicity was 2.70 and 0.38, respectively. Based on this finding, subsequent comparisons of cytotoxicity data from both assays were performed using IC_50_ values as well as for the drug’s corresponding MOS value.

In contrast, statistical comparison between the cytotoxicity data from both PHH and hLiMT assays for the 110 compounds set was challenging due to the disparate number of cytotoxicity IC_50_ values observed between assays and the overall number of compounds that did not have IC_50_ values converge. This difference made performing ROC curve assessments difficult, where only compounds with IC_50_ values could be included. This comparison would bias results towards cytotoxic compounds in both DILI+ve and DILI−ve classes, in turn misrepresenting the specificity in an assay-dependent manner due to the differences in the number of compounds with IC_50_ values obtained between the PHH and hLiMT. To demonstrate this limitation, we censored compounds that did not have IC_50_ values determined and then performed ROC curve analysis on IC_50_ or MOS values to determine the relative predictive value of each assay and the optimal threshold for DILI classification. Based on the optimized thresholds for both IC_50_ and MOS values, both PHH and hLiMT exhibited similar sensitivity for detection of known DILI causing drugs, with values between 63 and 72% (data not shown). Similarly, the specificity for both assays was similar, with values ranging between 50 and 57% for correct identification of non-DILI drugs when considering cytotoxicity IC_50_ values in isolation (data not shown). These findings were misleading, as there were 21 more IC_50_ values obtained for the test set in hLiMT assay relative to the PHH assay (Fig. 1; Supplemental Tables S2, S3).

### Statistical analysis employing practical fixed thresholds

In practice and in practical terms, the absence of a cytotoxicity signal (e.g., IC_50_ value) would indicate a negative signal. To account for this, we examined the performance of each assay by binary classification of all compound data into DILI+ve and DILI−ve groups with pre-defined thresholds based on practical cutoffs of 10, 25, 50, and 100 μM and 10×, 25×, 50×, and 100× for IC_50_ and MOS values, respectively. The summary for this analysis using IC_50_ values is presented in Table 2. Using this comparison, the sensitivity of hLiMT to identify DILI+ve compounds was greater at every threshold assessed in relation to PHH. For example, hLiMT identified 18.8% (13/69) of DILI+ve compounds in relation to 4.3% (3/69) determined by PHH using a 10 μM threshold (Table 2). Similarly at a 100 μM IC_50_ threshold, the sensitivity of hLiMT was twofold higher for hLiMT in relation to PHH assay, with values of 60.9% (42/69) and 33.3% (23/69), respectively (Table 2). Conversely, the specificity was high and similar across the assays over the four thresholds, with values ranging between 85 and 98% between the two assays. Using a 100 μM IC_50_ threshold, there were 6 false positives identified for both PHH and hLiMT assays, with 4/6 false positives consistent in both assays (digoxin, penbutolol, metergoline, and benztropine) (Supplemental Tables S2 and S3). The assay specificity observed in this comparison is in contrast to the specificity values obtained for PHH and hLiMT from the ROC curve assessment with the censored data, where values were 57 and 50%, respectively (data not shown). By incorporating all data in the statistical assessment, the specificity values are more in line with published reports of specificity of cytotoxicity assays to identify human hepatotoxicants that range between 70 and 90% (Dambach 2014; Schadt et al. 2015). When considering both sensitivity and specificity for hLiMT in relation to PHH with a 100 μM IC_50_ value threshold, hLiMT outperformed PHH with both higher PLR values (4.16 vs. 2.28) and lower NLR values (0.46 vs. 0.78) for this 110 compound set.

**Table 2:** Assay performance for PHH and hLiMT described based on pre-defined cytotoxicity IC_50_ thresholds

Assay	Number DILI+ve	Number DILI−ve	TP	TN	FP	FN	Threshold (μM)	Sensitivity (%)	Specificity (%)	PLR	NLR	Kappa	*P* value
2D PHH IC_50_ [μM]	69	41	3	40	1	66	10	4.3	97.6	1.78	0.98	0.014	0.61
	69	41	12	38	3	57	25	17.4	92.7	2.38	0.89	0.080	0.004
	69	41	20	37	4	49	50	29.0	90.2	2.97	0.79	0.177	0.007
	69	41	23	35	6	46	100	33.3	85.4	2.28	0.78	0.176	0.014
3D hLiMT IC_50_ [μM]	69	41	13	38	3	56	10	18.8	92.7	2.57	0.88	0.091	0.097
	69	41	26	36	5	43	25	37.7	87.8	3.1	0.71	0.215	0.004
	69	41	36	35	6	33	50	52.2	85.4	3.57	0.56	0.331	0.0001
	69	41	42	35	6	27	100	60.9	85.4	4.16	0.46	0.419	<0.0001

*TP* true positive, *TN* true negative, *FP* false positive, *FN* false negative, *PLR* positive likelihood ratio, *NLR* negative likelihood ratio, *Kappa* Cohen’s kappa concordance value

**Table 3:** Assay performance for PHH and hLiMT described based on pre-defined MOS thresholds

Assay	Number DILI+ve	Number DILI−ve	TP	TN	FP	FN	Threshold	Sensitivity (%)	Specificity (%)	PLR	NLR	Kappa	*P* value
2D PHH MOS (IC_50_/*C*_max_)	69	41	14	40	1	55	10×	20.3	97.6	8.32	0.82	0.141	0.008
	69	41	19	39	2	50	25×	27.5	95.1	5.64	0.76	0.183	<0.0001
	69	41	23	35	6	46	50×	33.3	85.4	2.28	0.78	0.156	0.031
	69	41	28	35	6	41	100×	40.6	85.4	2.77	0.70	0.221	0.004
3D hLiMT MOS (IC_50_/*C*_max_)	69	41	25	40	1	44	10×	36.2	97.6	14.86	0.65	0.279	0.0001
	69	41	33	38	3	36	25×	47.8	92.7	6.54	0.56	0.348	<0.0001
	69	41	36	35	6	33	50×	52.2	85.4	3.57	0.56	0.331	0.0001
	69	41	41	33	8	28	100×	59.4	80.5	3.05	0.50	0.363	<0.0001

*TP* true positive, *TN* true negative, *FP* false positive, *FN* false negative, *PLR* positive likelihood ratio, *NLR* negative likelihood ratio, *Kappa* Cohen’s kappa concordance value

### Statistical analysis incorporating margin of safety

The predictive value of cytotoxicity in hLiMT to identify clinical hepatotoxicants was further examined by evaluating the MOS for each compound as outlined above using fixed thresholds of 10×, 25×, 50×, and 100× MOS values. The summary of performance of each assay using this approach can be seen in Table 3. Similar to comparisons using IC_50_ values, the hLiMT experienced increased sensitivity to identify clinical hepatotoxicants when considering MOS values across all 4 thresholds evaluated (Table 3). For example at 10× MOS threshold, hLiMT assay experienced 36.2% (25/69) sensitivity in contrast to 20.3% (14/69) assay sensitivity of PHH in identifying DILI+ve compounds. In both assays at this 10× MOS threshold only 1 false positive (meclofenamate) was identified resulting in assay specificity of 97.6%. The PLR for PHH and hLiMT were 8.32 and 14.86, respectively. This indicates that an IC_50_ value obtained in PHH and hLiMT that is less than tenfold higher than the total plasma *C*_max_ values would cause a moderate or large increase the probability of clinical DILI, respectively. As the threshold increased to 25×, 50×, and 100×, higher PLR values and lower NLR values were consistently observed in hLiMT in relation to PHH (Table 3), supporting enhanced predictive value of hLiMT in relation to PHH to correctly identify clinical hepatotoxicants.

### Comparison of concordance of PHH and hLiMT with known DILI status

Concordance of binary classification of compounds as DILI+ve or DILI−ve determined by PHH or hLiMT assay IC_50_ or MOS values with the known clinical DILI categorization was assessed by estimating the kappa coefficient for each assay at each practical classification threshold (Tables 2, 3). Overall agreement with known-DILI status, as determined by Cohen’s kappa, was in general higher for the hLiMT than for the PHH when comparing within practical classification thresholds. Using MOS thresholds (Table 3), kappa values for the hLiMT assay were approximately twice that of kappa values for the PHH assay for each corresponding threshold. For both assays, the test of kappa = 0 is rejected at *α* = 0.05 for all thresholds, indicating that there is some agreement beyond random chance between the PHH assay and known DILI status, although the agreement between the hLiMT assay and known DILI status is stronger. Using IC_50_ thresholds (Table 2), the higher concordance of the hLiMT with known DILI status was more pronounced, especially at the lower thresholds tested. At the 10 μM IC_50_ threshold, neither assay showed statistically significant concordance with known DILI status beyond random chance agreement.

### Comparison of predictive value in PHH and hLiMT across different DILI categories

**Fig. 3:**
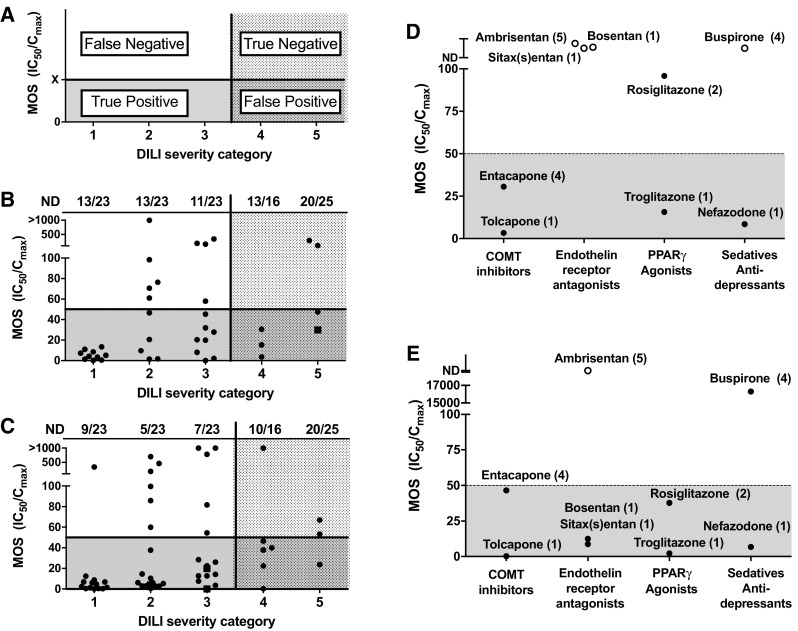
Exposure-corrected cytotoxicity (MOS) of 110 marketed drugs stratified across the five DILI severity categories. A compound was considered to be DILI+ve if classified in the following: DILI severity category 1 (Severe clinical DILI), severity category 2 (High clinical DILI concern, cases of liver failure), and severity category 3 (low clinical DILI concern, isolated and infrequent cases of DILI). Conversely, a drug was considered DILI−ve if classified as DILI severity category 4 (enzyme elevations in clinic) and severity category 5 (No DILI). **a** Schematic of the four quadrants identifying true and false positives and true and false negatives across the 5 DILI severity categories in relation to optimized MOS threshold. **b** Plated primary human hepatocytes, *dotted line* threshold of 50× MOS, **c** hLiMT, *dotted line* threshold of 50× MOS. DILI severity category is indicated in brackets with the number of compounds that did not have an IC_50_ value converge per DILI severity category listed at top. **d**, **e** Comparison of the MOS values for structurally related hepatotoxic and non-hepatotoxic compounds in hLiMT (**d**) and PHH (**e**) for catechol-O-methyltransferase (COMT) inhibitors (tolcapone and entacapone), endothelin-receptor antagonists (sitax(s)entan, bosentan and ambrisentan), insulin sensitizers (troglitazone and rosiglitazone), and sedatives/antidepressants (nefazodone and buspirone). *Filled circles* margin of safety (MOS) = IC_50_/*C*_max_ value; *open circles* not detected (ND) value and *filled square* <IC_50_/*C*_max_ value

As outlined above, hLiMT experienced greater sensitivity to identify compounds that were associated with clinical hepatotoxicity than PHH, regardless of comparing IC_50_ values alone or MOS calculations for binary DILI classification. The binary classification was necessary to identify the performance for each assay for identification of known clinical hepatotoxicants using fixed practical IC_50_ and MOS values as the thresholds for binning. However, this approach failed to provide detail on the predictive value of these assays for the compounds in each of the five DILI severity categories. MOS values and the number of compounds for which no IC_50_ value was detected (ND) for all compounds were plotted in relation to the five DILI severity categories with the 50× MOS threshold depicted for comparison (Fig. 3). The 50× threshold was plotted horizontally and in doing so, the true positives, false negatives, true negatives, and false positives for each assay can be visualized by quadrants (Fig. 3a). The numbers of false negative compounds (including both compounds above 50× MOS threshold and compounds with no IC_50_ value detected) for DILI severity category 1 compounds were lower for hLiMT (10/23) than PHH (13/23) (Fig. 3b, c). Similarly, the number of false negatives in DILI severity categories 2 and 3 were greater in the PHH in comparison to those identified in hLiMT assay. Interestingly, the hLiMT and PHH produced equal numbers (6) of false positive signals in DILI severity categories 4 and 5 using 50× MOS threshold. For example, the hLiMT detected only benztropine as a false positive from DILI severity category 5 in relation to the 3 false positive (digoxin, benztropine, procyclidine) from PHH in this category (Fig. 3b, c).

Further evaluation of the 14 day MOS values revealed that the hLiMT were able to better distinguish between structurally related hepatotoxic and non-hepatotoxic compounds than PHH for specific drug classes (Fig. 3d, e). In both PHH and hLiMT, catechol-O-methyl transferase (COMT) inhibitors entacopone and tolcapone fell below the MOS threshold of 50×. Both PHH and hLiMT were also able to correctly identify nefazodone as hepatotoxic and buspirone as not. However, PHH failed to classify any of the three endothelin-receptor antagonists as all compounds in this class did not have IC_50_ values. Conversely, hLiMT correctly identified sitax(s)entan and bosentan, both classified as DILI severity category 1, as positive with MOS values of 8.7× and 12.5×, respectively (Supplemental Table S-3). Moreover, hLiMT did not detect cytotoxicity and a corresponding MOS value for ambrisentan, which does not have clinical DILI associations (Fig. 3d, e). Similarly, hLiMT identified the peroxisome proliferator-activated receptor-gamma (PPARγ) agonists, troglitazone, and rosiglitazone, as DILI positive with MOS values less than 50×, while only troglitazone was detected in PHH with an MOS <50×.

### Time-dependent cytotoxicity and reproducibility of hLiMT assay

The head-to-head comparisons between PHH at 48 h treatment and hLiMT at 14 day treatment demonstrated increased predictive value of hLiMT to identify known hepatotoxicants in relation to PHH. It remained unknown whether the enhanced predictive value was due to differences between the complexity of culture or the differences in treatment duration across both assays. To begin to address this, we evaluated cytotoxicity of a subset (38) of the 110 compounds in hLiMT treated for 5–6 days in relation to the cytotoxicity in 14-day treatment. Dose and time-dependent cell toxicity was observed with compounds for which cell viability was determined after 5–6 days or 14 days exposure. Prolonged exposure resulted in decreased IC_50_ values for 21 out of 38 compounds where data were obtained following both 5–6 and 14 day exposure. (Supplementary Figure S2; Supplementary Table S4). A higher IC_50_ value was observed with only 1/38 drugs (flutamide, *n* = 1), all other values were unaffected. This data support that the treatment duration is a significant contributor to achieve lower IC_50_ values across most of the test set (Fig. 1a).

**Fig. 4:**
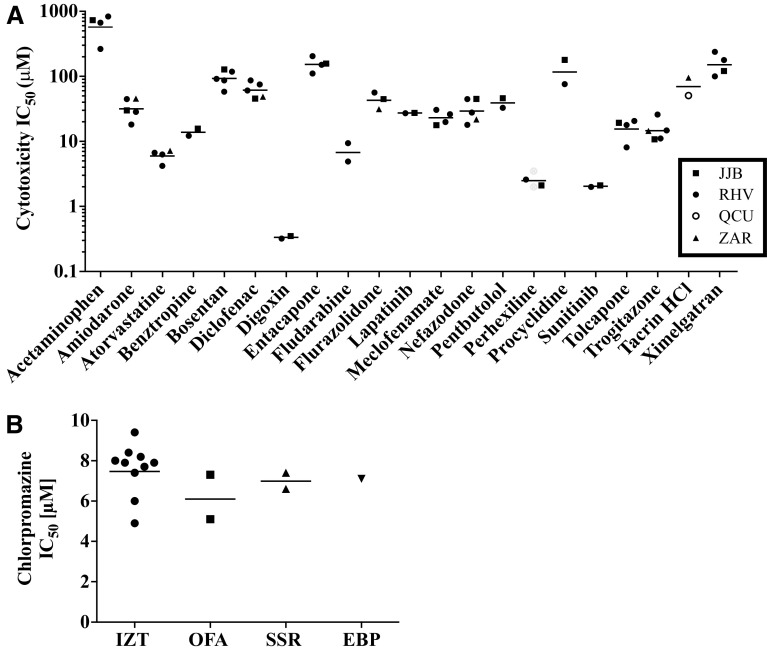
Reproducibility of the IC_50_ values from independent experiments following incubation of the 3D hLiMT to test compounds for 14 days. Cytotoxic IC_50_ values for subset of compounds were plotted for hLiMT were prepared using either: the same cryopreserved primary human hepatocyte lot (IZT) but four different NPC lots: *filled square* JJB; *filled circle* RHV; *filled triangle* ZAR; *open circle* QCU (**a**) or the same NPC lot but different lots of cryopreserved primary human hepatocytes (IZT, OFA, SSR or EBP) (**b**)

Care was taken to evaluate cytotoxicity for the 110 drugs in hLiMT and PHH using identical hepatocyte lots from the same donor to ensure that donor-to-donor variability would not affect interpretation of the results. However, the effect of the NPC donor on the enhanced sensitivity/specificity observed by the hLiMT in relation to PHH in identifying known hepatotoxicants could not be ruled out. To address this, we compared the 14 day IC_50_ values obtained from 2 to 5 independent experiments with hLiMT prepared with different NPC lots and a fixed hepatocyte source for 21 drugs. The data (presented in Fig. 4; Supplemental Table S3) revealed that the data obtained from a single donor of hepatocytes was reproducible and unaffected by preparation of microtissues using different NPC lots (Fig. 4a). In addition, comparison of the chlorpromazine IC_50_ values obtained following incubation of hLiMT prepared using the same NPC lot, but different PHH donors (*n* = 1–10), revealed only minor changes in the IC_50_ values (Fig. 4b).

### Measurement of exploratory biomarkers of liver injury in the hLiMT assay

**Fig. 5:**
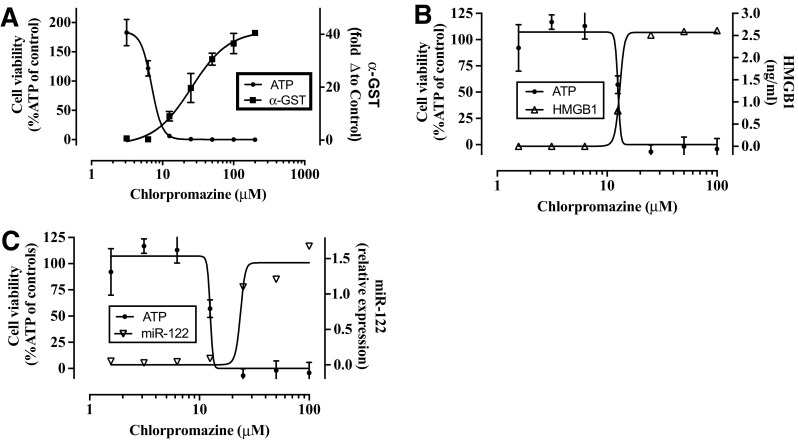
POC study demonstrating the utility of hLiMTs to detect biomarkers of hepatocellular injury in vitro. Representative changes in biomarker levels are depicted following exposure to chlorpromazine for 14 days (**a**) or 5 days (**b** and **c**). **a** In relation to decreases in total cellular ATP (*filled circle*) as determined on day 14. *Filled square α*-GST levels were determined on days 5, 9, and 14 and the values combined to give a fold change relative to control. **b**, **c** In relation to decreases in total cellular ATP as determined on day 5. *Filled circle* cell viability (% control); **b** *open triangle* HMGB-1 ng/ml, **c** *open inverted triangle* miRNA-122 relative expression. Data are from single experiments in triplicate

A potential limitation of the hLiMT assay was that the spheroids were comprised of only approximately 1000 cells, which could limit the sensitivity to detect secondary endpoint measurements in the supernatant such as exploratory and mechanistic biomarkers of liver injury. As a proof of concept, we evaluated the dose- and time-dependent release of *α*-GST, total levels of HMGB1, and relative expression of miR-122 into the cell-culture supernatant of individual spheroids treated with a subset of compounds (Supplemental Methods). Dose and time-dependent release of *α*-GST into the hLiMT supernatant was observed for 8/9 DILI compounds where a toxic response was elicited, with the release correlating well with the observed decreases in intracellular ATP. In contrast, no release of *α*-GST into the hLiMT supernatant was observed for 5 non-DILI compounds (Supplementary Table S5). An example of the observed release of *α*-GST and the depletion of ATP following exposure to chlorpromazine for 14 days can be found in Fig. 5a. Similarly, dose-dependent release of the miR-122 and HMGB1 were also observed following exposure of the hLiMT to chlorpromazine for 5 days (Fig. 5b, c). As with the release of *α*-GST, the release of these biomarkers also correlated well with the observed decreases in intracellular ATP levels. Together, this data supported that mechanistic and exploratory biomarkers could readily be detected in the supernatant of the hLiMT assay in response to drug-induced cytotoxicity.

## Discussion

A significant challenge associated with identifying hepatotoxicity risk in drug discovery is that in vivo studies in preclinical species have poor concordance of identifying human related hepatotoxicity (Olson et al. 2000). Major efforts have been undertaken in order to improve prediction of potential hepatotoxic drugs without utilizing animal testing. In preclinical settings in vitro cell-based assay methods are frequently used to test DILI potential of drugs (reviewed in Chen et al. 2014), as these assays enable the monitoring of a cellular response after drug exposure. They also provide the possibility of high throughput screening and have a low requirement for quantity of drug substances. However, the ability of these assays to detect parent compound and metabolite mediated cytotoxicity is significantly limited as not all of the cell-based systems employed contain the full complement or functionality of metabolic enzymes and transporters present in human hepatocytes (Gustafsson et al. 2014; Wilkening and Bader 2003) and plated primary human hepatocytes rapidly lose liver phenotype and CYP450 activity in traditional monolayer cultures (Rodriguez-Antona et al. 2002; Rowe et al. 2013). 3D spheroid models are reported to produce more accurate assessment of acute and possibly also chronic drug-induced hepatotoxicity than traditional 2D culture models (Messner et al. 2013) and to be capable of detecting compounds with cholestatic liability (Hendriks et al. 2016). Moreover, 3D multicellular liver spheroids require low cell numbers (e.g., 500–5000 cells), express relevant transporters, maintain functionality over 28 days in culture, and can be produced in a 96-well format (Messner et al. 2013), which make them amenable to higher throughput long-term repeat-dose testing in early discovery.

Here, we present the findings of a comprehensive evaluation of a high throughput 3D human liver spheroid (hLiMT) assay for retrospective prediction of clinical hepatotoxicity versus 2D PHH, which are considered to be the ‘gold standard’ for human hepatotoxicity assessment (LeCluyse 2001). We demonstrated that a single cell-health endpoint on 3D primary hepatocyte co-cultures after a 14-day drug exposure is sufficient for prediction of DILI with modest/moderate sensitivity (19–61%) and high specificity (81–98%) depending on thresholds employed. This is important since multi-parameter approaches measuring sublethal pathways are often cost-prohibitive, with large screening needs for early discovery support and medicinal chemistry design requirements based on structure toxicity relationships (Shah et al. 2015). Hence, the use of simplified assays that highlight intrinsic risks are preferred over high-content screening approaches since this can miss identification of pathways that are time-dependent, influenced by hermitic responses and do not necessarily identify a mechanism of toxicity but highlight a cell injury pathway. The more costly high-content approaches can then be reserved for deployment at appropriate situations within the drug discovery pipeline where increased mechanistic insight is desirable.

The direct comparison of 110 drugs on 3D hLiMT and 2D PHH cultures resulted in a clear difference in usefulness of the model systems for prediction of DILI. Expression of the 14 day ATP IC_50_ values relative to the human plasma total *C*_max_ concentration enabled determination of a “margin-of-safety” (MOS; Supplementary Tables S2,S3). The initial goal of the analysis was to identify an optimal threshold for IC_50_ and MOS values that would best separate the known hepatotoxicants from those without clinical DILI; it became clear that this metric would be highly dependent on the compound set employed, concentration ranges achievable in vitro, and accordingly the number of IC_50_ or MOS values obtained from the test set for each assay. As such, we presented the predictive value of PHH and hLiMT assays based on practical thresholds for both cytotoxicity IC_50_ values as well as MOS values that would likely be implemented in drug discovery at different stages of lead-optimization. Across this comparison, hLiMT assay experienced greater sensitivity and equivalent specificity to PHH in distinguishing between known DILI compounds and non-DILI compounds. In general, the overall agreement with known-DILI status, as determined by Cohen’s kappa, was higher for the hLiMT than for the PHH when comparing within practical classification thresholds. The observed increased sensitivity in the 3D hLiMT versus the traditional 2D culture model is consistent with reports that 3D spheroid models produce better risk assessment of drug-induced hepatotoxicity (Gunness et al. 2013). In addition, we found that long-term exposure of the 3D hLiMT resulted in enhanced sensitivity for the detection of DILI positive drugs versus short-term culture (Figure S1; Supplementary Table S4). These data are in agreement with the findings of Bell et al. (2016) who noted prolonged drug exposure of up to 28 days resulted in increased sensitivity for detection of DILI compounds amiodarone, bosentan, diclofenac, fialuridine, and tolcapone (Bell et al. 2016). Moreover, we also demonstrated that total plasma *C*_max_ alone was a good predictor of potential DILI risk. Using this dataset, human total plasma *C*_max_ threshold of 1.3 µM (Fig. 2) distinguished DILI positive from DILI negative compounds with a sensitivity of 73% and a specificity of 73%. These data back up similar reports by Shah et al. (2015) who also demonstrated that a *C*_max_, total threshold of 1.1 µM was a major driver in distinguishing DILI-positive and DILI-negative compounds (sensitivity/specificity 80/73%). In both these studies, and the study by Shah et al. (2015), incorporating the plasma total *C*_max_ values improved the sensitivity/specificity for each assay and helped to derive predictive margins of safety.

The hLiMT assay was also able to retrospectively distinguish between matched pairs of drugs, with the MOS values for the non-hepatotoxic drugs ambrisentan and buspirone falling above the threshold value of 50×, and the MOS values for their hepatotoxic structural analogues bosentan, sitax(s)entan and nefazadone, falling below the threshold value (Fig. 3e). In particular, the findings highlight an increased sensitivity of hLiMT to the cytotoxicity from the hepatotoxic endothelin-receptor antagonists, bosentan and sitax(s)entan, in relation to PHH, where no IC_50_ values were detected. Both drugs have strong association with DILI, where bosentan has been given a cautionary “black box” warning for DILI by the FDA and sitax(s)entan was voluntarily removed from the market due to hepatotoxicity concerns. Extensive studies, in particular on bosentan, support potential mechanisms of BSEP transport inhibition, and mitochondrial toxicity that lead to intrahepatic cholestasis and hepatocellular injury (Fattinger et al. 2001; Kenna et al. 2015). It remains unclear if the treatment time, enhanced liver phenotype, or presence of bile-canicular membranes were responsible for the increased sensitivity of hLiMT to these compounds relative to PHH in our studies. However, a recent report suggested that bile-acid transport inhibition might be involved in part in bosentan-induced cytotoxicity in this model. Addition of extracellular bile acids in the cell-culture media caused increased cytotoxicity of liver spheroid cultures relative to normal media treated spheroid cultures treated with bosentan over 14 day (Hendriks et al. 2016). In our studies, ambrisentan was not cytotoxic to hLiMT and has a reported 10- and 30-fold lower potency to inhibit BSEP transporter function than bosentan and sitax(s)entan, respectively (Kenna et al. 2015). Taken together, the findings in this report in addition to the recent published report by Hendriks et al. (2016) support that hLiMT may be a valuable in vitro tool to evaluate the functional and phenotypic (e.g., cytotoxicity) effects of bile-acid transport inhibition in an intact hepatocellular model. Considering that mechanisms leading to alterations in bile-acid homeostasis are believed in part to be responsible for recent prominent late-stage clinical attritions and black box warnings of novel therapeutics, including examples such as CP-724,714 (Feng et al. 2009), tolvaptan (Slizgi et al. 2016), AMG-009 (Morgan et al. 2013), and TAK-875 (Wolenski et al. 2017), there is an increasing need and awareness to better characterize the phenotypic effects of bile-acid inhibition in drug discovery. Accordingly, continued characterization of spheroid hepatic models in regards to bile-acid synthesis, transport, and homeostasis and their effects to drug treatment is warranted.

Although hLiMT out performed PHH in identifying hepatotoxicants, the assay failed to properly classify approximately 40% of the DILI+ve compounds tested. In particular, no cytotoxicity was observed with 23/69 DILI+ve drugs (Figs. 1, 3) and when correcting for clinical exposure 28/69 DILI+ve compounds fell above an MOS value of 100x (Table 3). This is not surprising in that DILI is comprised of many different etiologies and mechanisms, including factors that are both compound- and patient-related (Chalasani and Bjornsson 2010; Chalasani et al. 2008). Many of the compounds falsely classified were associated with low or very low incidence of DILI within the patient population and are considered idiosyncratic hepatotoxicants and difficult to identify using individual assays in isolation. For example rosuvastatin is associated with mild, transient elevations (1–3%) of plasma enzyme levels with acute liver injury only occurring in 1 in 10,000 patients (Russmann et al. 2005). Accordingly, there are several compelling reports demonstrating high predictive value using multi-parametric approaches to identify known hepatotoxicants, many of which are considered idiosyncratic (Aleo et al. 2014; Schadt et al. 2015; Shah et al. 2015; Thompson et al. 2012). These retrospective studies support that although the clinical manifestation of DILI for many drugs may appear idiosyncratic, there does appear to be intrinsic properties of the molecules that pose risk for hepatotoxicity. In this vein, the hLiMT assay appears to be an additional tool to add to the suite of in silico, in vitro, and in vivo studies used to characterize hepatotoxicity risk in drug discovery and can be positioned differently by each institution based on their level of risk tolerance, throughput needs, and other considerations.

In conclusion, spheroid hepatic cultures experienced greater mechanistic coverage and sensitivity, while maintaining similar specificity to the standard PHH assay. The hLiMT demonstrated sufficient reproducibility across studies and across different preparations with cells isolated from multiple donors. In addition, the potential of the 3D hLiMT to report on the release of novel translational in vivo liver hepatotoxicity biomarkers, miR-122, a highly liver specific microRNA (Wang et al. 2009), HMGB1, a marker of immune modulation and necrosis (Antoine et al. 2009), and *α*-GST, a sensitive, highly specific and early biomarker for hepatocellular injury (Muller and Dieterle 2009), following exposure to DILI positive drugs demonstrated in principle that this 3D liver model has the potential to recapitulate in vivo findings in vitro (Fig. 5; Supplementary Table S-4). Taken together, the data produced in this comprehensive evaluation of the 3D hLiMT model support that hLiMT outperformed PHH in identifying clinically relevant hepatotoxicants when measuring cytotoxicity as an endpoint. This is an important finding considering that hepatotoxicity remains a major source of clinical drug attrition and post-market withdrawal of drugs (Watkins 2011). Therefore, alongside other recently published studies, this study supports the use of hepatic spheroid models to aid hepatotoxicity risk assessment in drug discovery.

## Electronic supplementary material

Below is the link to the electronic supplementary material.

Supplementary material 1 (PDF 322 kb)

Supplementary material 2 (PDF 333 kb)

Supplementary material 3 (XLSX 166 kb)

Supplementary material 4 (DOCX 18 kb)

## Abbreviations

ALI: Acute liver injury
*α*-GST: Alpha-glutathione-s-transferase
2D: Two-dimensional
3D: Three-dimensional
DILI: Drug-induced liver injury
ELISA: Enzyme-linked immunosorbent assay
hLiMM: Human liver microtissue maintenance medium
hLiMT: Human liver microtissue
HMGB1: High mobility group box 1
NLR: Negative likelihood ratio
NPC: Non-parenchymal cells
PHH: Primary human hepatocytes
P450: Cytochrome P450
miR-122: MicroRNA-122
MOS: Margin-of-safety
qPCR: Quantitative polymerase chain reaction
PLR: Positive likelihood ratio
ROC: Receiver operating characteristic analysis
RT PCR: Real-time PCR
ULN: Upper limit of normal
