# Supplementary material for: Utility of spherical human liver microtissues for prediction of clinical drug-induced liver injury
Source: Arch Toxicol. 2017 Jun 13;91(8):2849–63. doi: 10.1007/s00204-017-2002-1 (PMC5515971; doi:10.1007/s00204-017-2002-1)
Supplement: Supplementary file 1 — Supplementary material 1 (PDF 322 kb) [file 204_2017_2002_MOESM1_ESM.pdf]

Utility of Spherical Human Liver Microtissues for Prediction of Clinical Drug-Induced Liver Injury

Proctor, WR[2], Foster,AJ[1†], Vogt, J[2], Summers, C[1], Middleton, B[1], Pilling, MA[1], Sheinson,D[2], Kijanska,M[3], Ströbel, S[3], Haugstetter, J[3], Kelm, JM[3], Misner, D[2], Morgan, P[1], Messner, S[3], Williams, D[1]

[1] Drug Safety and Metabolism, †Discovery Sciences, AstraZeneca, Alderley Park, Macclesfield, Cheshire, SK10 4TG, and Cambridge Science Park, Cambridge, Cambridgeshire, CB4 0WG, United Kingdom
[2] Investigative Toxicology, Department of Safety Assessment, Genentech, Inc. 1 DNA Way, South San Fransisco, CA, 94080, USA
[3] InSphero AG, Wagistrasse 27, 8952 Schlieren, Switzerland

[†]Corresponding author: Alison Foster, Drug Safety and Metabolism, AstraZeneca, Cambridge Science Park, Cambridge, Cambridgeshire, CB4 0WG, United Kingdom email address: alison.foster2@astrazeneca.com

Supplemental Figure: S1

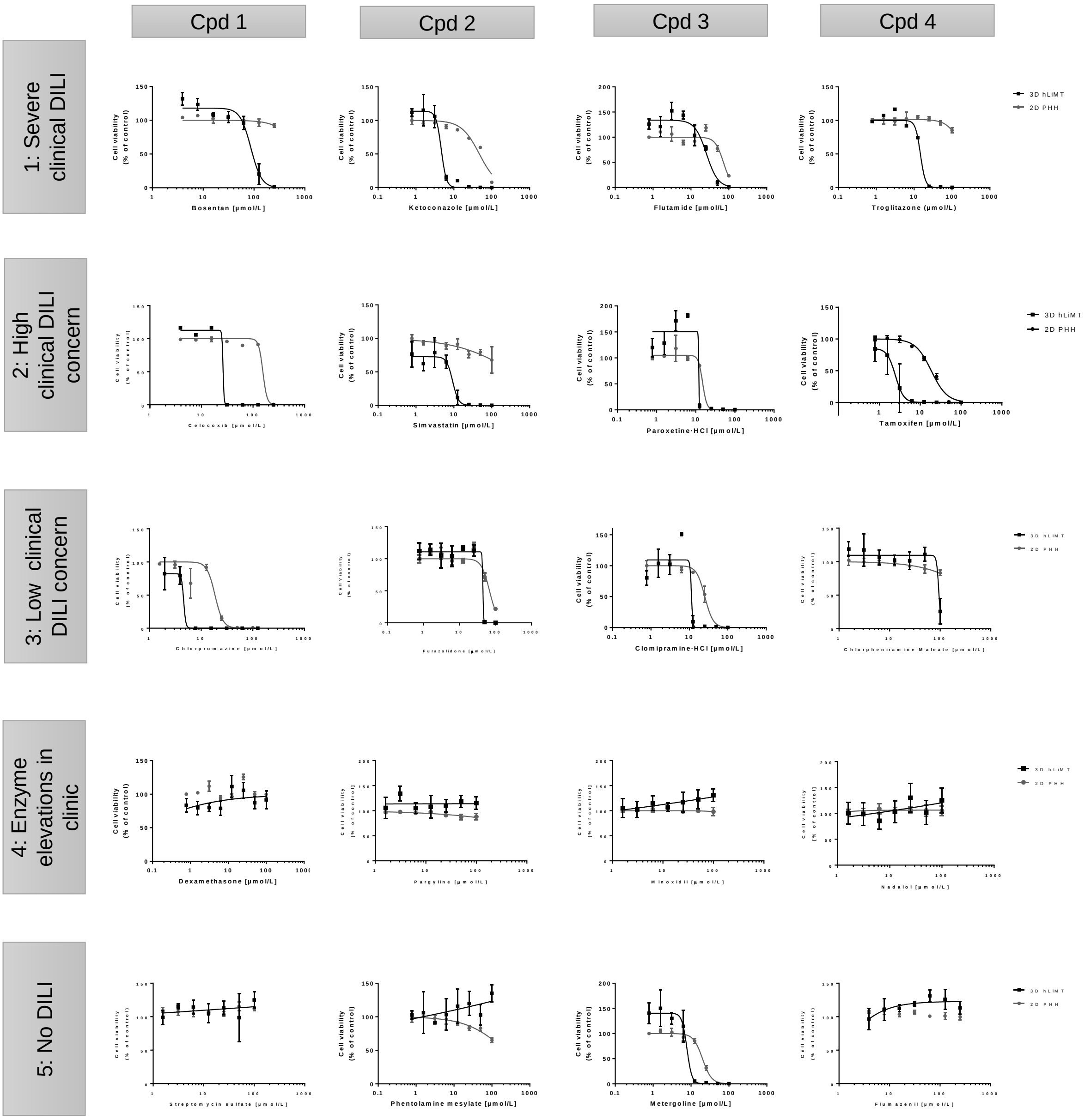

Supplemental Fig. S1. Exemplary graphs (randomly chosen) of dose-response curves obtained from 2D PHH (●) and 3D hLiMT (■). Four compounds are displayed from each DILI-severity category.
