# Supplementary material for: Utility of spherical human liver microtissues for prediction of clinical drug-induced liver injury
Source: Arch Toxicol. 2017 Jun 13;91(8):2849–63. doi: 10.1007/s00204-017-2002-1 (PMC5515971; doi:10.1007/s00204-017-2002-1)
Supplement: Supplementary file 4 — Supplementary material 4 (DOCX 18 kb) [file 204_2017_2002_MOESM4_ESM.docx]

**Utility of Spherical Human Liver Microtissues for Prediction of Clinical Drug-Induced Liver Injury**

Proctor, WR^2^, Foster,AJ^1†^, Vogt, J^2^, Summers, C^1^, Middleton, B^1^, Pilling, MA^1^, Sheinson,D^2^, Kijanska,M^3^, Ströbel, S^3^, Haugstetter, J^3^, Kelm, JM^3^, Misner, D^2^, Morgan, P^1^, Messner, S^3^, Williams, D^1^

^1^ Drug Safety and Metabolism, †Discovery Sciences, AstraZeneca, Alderley Park, Macclesfield, Cheshire, SK10 4TG, and Cambridge Science Park, Cambridge, Cambridgeshire, CB4 0WG, United Kingdom

^2^ Investigative Toxicology, Department of Safety Assessment, Genentech, Inc. 1 DNA Way, South San Fransisco, CA, 94080, USA

^3^ InSphero AG, Wagistrasse 27, 8952 Schlieren, Switzerland

^†^Corresponding author: Alison Foster, Drug Safety and Metabolism, AstraZeneca, Cambridge Science Park, Cambridge, Cambridgeshire, CB4 0WG, United Kingdom email address: alison.foster2@astrazeneca.com

**Supplementary methods**

## Reagents and Chemicals

The following reagents were obtained from Life Technologies Ltd (Paisley, United Kingdom): Taqman microRNA (RT) kit. The alpha-glutathione-S-transferase (α-GST) ELISA kit was obtained from EKF Diagnostic, Dublin Ireland. The high mobility group box 1 (HMGB1) ELISA kit was obtained from IBL International, Hamburg, Germany. RT primers were obtained from Applied Biosystems, Foster City, CA. The QIAzol lysis reagent, the miRNeasy^R^ mini kit (50) and the RNeasy MinElute Cleanup Kit were obtained from QIAgen, Manchester, UK. All other reagents were obtained from Sigma-Aldrich Company Ltd (Poole, Dorset, UK) or SigmaAldrich, St. Louis, MO, USA.

## Determination of Alpha-Glutathione S-Transferase (α-GST) activity in hLiMT supernatants

Supernatants from single hLiMT (in triplicates) were analysed for α-GST content using EKF’s α-GST ELISA assay. Briefly, 50 μL of the thawed cell-culture supernatants from single microtissues was transferred into a new v-bottom plate and diluted with 60 μl sample diluent buffer. From this dilution, 100 μL were transferred to the microassay strips. The ELISA was otherwise performed according to the manufacturer’s instructions.

## Determination of High Mobility Group Box 1 (HMGB1) levels in hLiMT supernatants

Supernatants from single hLiMT (in triplicates) were analysed for HMGB1 content using IBLs ELISA assay using the high sensitive range test procedure. Briefly, 50 μL of the thawed cell-culture supernatants from single microtissues or diluted standards were incubated in the microtiter plate along with 50 μL of the diluent buffer for 20-24 h at 37 ^o^C in the dark. The plate was then washed 5 times with 400 µL diluted wash buffer (1:5), blotted dry and 100 µL enzyme conjugate added. After incubation in the dark for 2 h at 25 ^o^C the plate was washed 5 times with 400 µL diluted wash buffer, blotted dry and then 100 µL colour solution added. The plate was incubated for a further 30 min at room temperature prior to the addition of 100 µL stop solution. The absorbance was then determined at 450 nm using an EnVision^®^ plate reader (PerkinElmer, Waltham, MA, USA) within 60 min. The concentration of HMGB1 in the samples was read off the high sensitivity range standard curve obtained from plotting the OD of the standards against the concentration (ng/L).

## Determination of miR-122 levels in hLiMT supernatants

**miRNA extraction** miRNA was extracted using a miRNeasy kit, following the manufacturer's instructions, and as described by Starkey et al. 2016 with minor modifications. Thirty-eight µL thawed hLiMT supernatant was mixed with 162 µL nuclease-free water and 700 µL QIAzol reagent and then incubated for 5 min at room temperature. Chloroform (100 µL) was added, the samples shaken vigorously for 15 sec and then centrifuged at 12,000 g for 15 min at 4^o^C. The supernatant was then mixed with 500 µL 70% ethanol and transferred to a miRNeasy minispin column. The column was centrifuged at >8000 g for 15 sec. The elute was then purified using a MinElute kit

**miRNA purification** The small RNA elution mixture was applied to the MinElute column 700 µL at a time. The immobilized RNA washed with various buffers before a final wash with 80% ethanol. The column was then dried by centrifugation. The small RNA fraction was eluted in 14 µL nuclease-free water and stored frozen at -80 ^o^C.

**RT PCR** Complementary DNA (cDNA) was synthesised from total RNA using the Taqman microRNA (RT) kit in a total reaction volume of 15 μL Six µl thawed RNA sample was mixed with 9 µl master mix containing 0.5 µL 100 mM dNTP, 1 µl 50 U/µL Multiscribe^TM^ reverse transcriptase, 1.5 µL 10X RT buffer, 0.19 µL 20U/µL Rnase inhibitor, 4.66 µL nuclease-free water, 0.75 µL miR-122 20X RT primer, 0.75 µL Cel-lin4 20X RT primer in a well optical plate. Following incubation on ice for 5 min, the samples were incubated in a DNA engine thermal cycler (MJ Research). The qRT-PCR thermal cycling program consisted of an initial 30 min incubation at 16 ^o^C, 30 min at 42 ^o^C, 5 min at 85 and then 4 ^o^C for infinity. The cDNA was then stored at -80 ^o^C.

**qRT-PCR** One µl thawed cDNA was mixed with 19 µl miR-122 or cel-lin4 PCR reaction master mix containing 1 µL Taqman^TM^ miR-122 or cel-lin4 primer small RNA assay (20X), 10 µl Taqman^TM^ universal PCR master mix II (2X) and 8 µL nuclease free water. Levels of miRNA were measured by the fluorescent signal produced from the Taqman probes on an ABI Prism 7000 (Applied Biosystems). miRNA levels were normalized to levels of cel-lin4 miRNA. Relative miRNA production was determined with the ΔCt method and reported as 2−ΔCt, where Ct is the threshold cycle. Differences in miRNA concentration were expressed as fold changes relative to control.
